# Implications of an Underlying Beckwith–Wiedemann Syndrome for Wilms Tumor Treatment Strategies

**DOI:** 10.3390/cancers15041292

**Published:** 2023-02-17

**Authors:** Paola Quarello, Diana Carli, Davide Biasoni, Simona Gerocarni Nappo, Carlo Morosi, Roberta Cotti, Emanuela Garelli, Giulia Zucchetti, Manuela Spadea, Elisa Tirtei, Filippo Spreafico, Franca Fagioli

**Affiliations:** 1 Pediatric Onco-Hematology, Stem Cell Transplantation and Cellular Therapy Division, Regina Margherita Children’s Hospital, 10126 Turin, Italy; 2 Department of Public Health and Pediatrics, University of Turin, 10124 Turin, Italy; 3 Immunogenetics and Transplant Biology Service, Città della Salute e della Scienza University Hospital, 10126 Turin, Italy; 4 Department of Medical Sciences, University of Turin, 10124 Turin, Italy; 5 Pediatric Surgical Oncology Unit, Fondazione IRCCS Istituto Nazionale dei Tumori, 20133 Milan, Italy; 6 Pediatric Urology Unit, Regina Margherita Children’s Hospital, 10126 Turin, Italy; 7 Department of Radiology, Fondazione IRCCS Istituto Nazionale dei Tumori, 20133 Milan, Italy; 8 Pediatric Radiology, Regina Margherita Children’s Hospital, 10126 Turin, Italy; 9 Pediatric Oncology Unit, Department of Medical Oncology and Hematology, Fondazione IRCCS, Istituto Nazionale dei Tumori, 20133 Milan, Italy

**Keywords:** Beckwith–Wiedemann syndrome, Wilms tumor, nephron-sparing surgery

## Abstract

**Simple Summary:**

Beckwith–Wiedemann Syndrome (BWS) is one of the five most frequent syndromes predisposing to Wilms tumor (WT). BWS can be underdiagnosed if the phenotype is mild, and WT can be the presenting symptom. The purpose of this review is to summarize the available data in the current literature on the diagnosis, surveillance, treatment strategies, and outcome of WT in the presence of BWS. The awareness of clinical and pathological features of WT suggestive of BWS-associated WT can lead to prompt genetic counselling and, consequently, to the most appropriate treatment.

**Abstract:**

Beckwith–Wiedemann Syndrome (BWS) is a pediatric overgrowth disorder involving a predisposition to embryonal tumors. Most of the tumors associated with BWS occur in the first 8–10 years of life, and the most common is Wilms tumor (WT). BWS clinical heterogeneity includes subtle overgrowth features or even silent phenotypes, and WT may be the presenting symptom of BWS. WT in BWS individuals exhibit distinct characteristics from those of sporadic WT, and the management of these patients needs a peculiar approach. The most important feature is a higher risk of developing bilateral disease at some time in the course of the illness (synchronous bilateral disease at diagnosis or metachronous recurrence after initial presentation with unilateral disease). Accordingly, neoadjuvant chemotherapy is the recommended approach also for BWS patients with unilateral WT to facilitate nephron-sparing surgical approaches. This review emphasizes the importance of early BWS recognition, particularly if a WT has already occurred, as this will result in an urgent consideration of first-line cancer therapy.

## 1. Introduction

The most frequent renal malignancy in childhood is Wilms tumor (WT), also known as nephroblastoma, which accounts for 7% of all pediatric cancers [1]. A WT-predisposing syndrome, a malformation syndrome, or a germline (epi)mutation is present in more than 10% of WT patients [2,3,4,5,6,7]. One of the syndromes most frequently linked to WT is Beckwith–Wiedemann syndrome (BWS, OMIM 130650), an inborn overgrowth disease brought on by molecular changes in chromosome 11p15.5 [8,9]. Its clinical presentation varies greatly from case to case, with some patients lacking classical characteristics and showing only mild phenotypes. In order to better reflect the variety of the clinical symptoms that may appear in BWS patients, the syndrome was renamed Beckwith–Wiedemann Spectrum [9,10]. Furthermore, BWS demonstrates etiologic molecular heterogeneity with specific phenotype–epi/genotype associations. The overall risk of embryonal tumors in BWS patients has been estimated in the range of 0.2–24%, depending on the underlying genetic cause. WT is the most frequently reported tumor, affecting 1–8% of individuals. Other tumors include hepatoblastoma, neuroblastoma, rhabdomyosarcoma, and adrenal carcinoma [11].

WT in BWS individuals may exhibit distinct characteristics from non-syndromic WT, such as a higher likelihood of bilateral synchronous disease or the development of metachronous WTs, which demand a focused clinical management.

This review summarizes data on the diagnosis, surveillance, treatment strategies and outcome of WT in the presence of BWS from the up-to-date literature.

## 2. Material and Methods

PubMed, Embase, Web of Science were searched for relevant articles, using the following key terms: “Wilms tumor”, “Beckwith Wiedemann Syndrome”, “Nephrogenic rests”, “Nephroblastomatosis”, “Bilateral Wilms tumor”, “Wilms tumor predisposing syndromes”, “Wilms tumor predisposing conditions” “Nephron sparing surgery”. Studies published in a language other than English were excluded. The search allowed to identify the main data on the implications of BWS for WT treatment strategies described in the present review.

## 3. Results

### 3.1. WT Predisposing Conditions

Wilms tumor, aniridia, genitourinary anomalies, mental retardation (WAGR) syndrome, Denys–Drash syndrome (DDS), BWS, isolated hemihypertrophy (HHI), and Pearlman syndrome are the syndromes most frequently linked to WT [4,12]. These conditions show different levels of risk for malignancies. Scott et al. defined a classification of these syndromes in three different oncologic risk categories [4]. WAGR, DDS, familial Wilms tumor, Pearlman syndrome, mosaic variegated aneuploidy, and Fanconi anemia D1 (biallelic BRCA2 mutations) are among the syndromes with a high risk (>20%) of developing WT. BWS, Fraiser syndrome, and Simpson Golabi–Behemel syndrome are included in the moderate risk group (5–20%). Other syndromes such as HHI, Bloom syndrome, Li–Fraumeni syndrome/Li–Fraumeni-like syndrome, hereditary hyperparathyroidism–jaw tumor syndrome, Mulibrey nanism, Trisomy 18, and 2q37 microdeletion syndrome are associated with a risk lower than 5% of developing WT [4,13,14].

An increasing number of WT predisposition genes has been identified due to recent progresses in genomic assessment. The Factors Associated with Childhood Tumors Study was the largest study on germline variants in pediatric solid tumor, including 799 patients with non-familial WT [15]. With these latest analyses, there are now 21 germline mutated genes that have been validated as WT predisposition genes.

Molecular alterations in *WT1*, *TRIM28*, *REST*, and 11p15 epimutations and uniparental disomy that result in biallelic IGF2 expression are the most frequent and contribute to about 8% of unselected WT. The remaining 17 genes are quite uncommon and collectively contribute to 2% of unselected WT only [15].

Moreover, Hol et al. found that 33% of a large cohort of 126 unselected WT patients exhibited germline alterations, which was significantly higher than expected. The most frequently involved genes/loci were WT1 (7.9%) and 11p15 (15.9%) [7].

Indeed, it is likely that the known prevalent (epi)genetic predisposing alterations represent the tip of the undiscovered iceberg [7,16]. There are different attitudes towards referring children with WT to a clinical geneticist. Some authors encourage standard genetic testing after counseling for all patients with WT [7], despite nearly two-thirds of affected children not having an identified genetic predisposition, and many hospitals do not have enough resources for such a universal strategy. 

**Figure 1:**
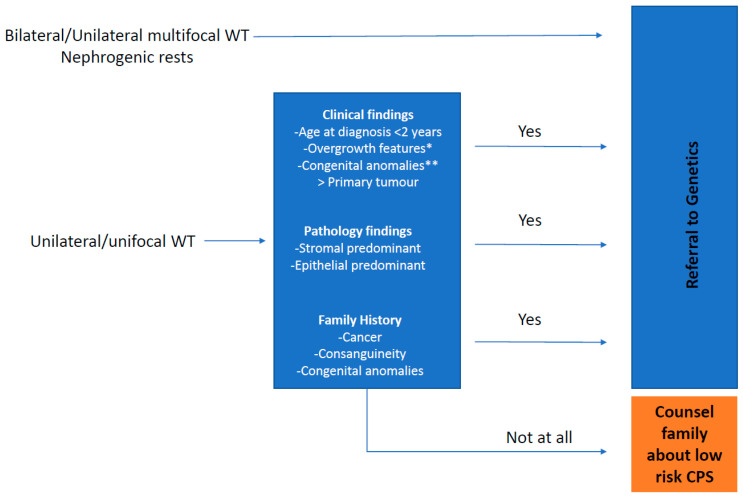
Decision tree for cancer genetics referral. * Macroglossia, lateralized overgrowth, macrosomia, macrocephaly, tall stature. ** aniridia, genitourinary abnormalities, nephropathy, cardiac malformations, ear anomalies, abdominal wall defects, polyhydramnios, dysmorphic facial features, café au lait spots, nevus flammeus, axillary/inguinal freckling. CPS: cancer predisposition syndrome [Adapted from [7,16,17]].

Others recommend using evidence-based algorithms to prioritize genetic testing for only a limited number of selected patients [16]. Turner et al. described their decision-support guide for selective referral of patients with WT for a cancer genetic evaluation, detailing risk clinical factors for cancer genetic predisposition syndrome, also considering mild factors. The most important ‘red flags’ that were recognized as suggestive of a WT genetic predisposition were: bilaterality/multifocality, presence of nephrogenic rests (NRs), young age at tumor onset, and presence of overgrowth features [7,17,18,19]. (Figure 1). 

### 3.2. Beckwith–Wiedemann Syndrome

#### 3.2.1. Clinical Aspects

BWS patients present a wide clinical spectrum of features including several variably associated anomalies; some mild phenotypes lack typical hallmarks, which causes the prevalence of this disease to be underestimated. The features of the classical BWS besides overgrowth include abdominal wall defects (omphalocele, umbilical hernia, or diastasis recti), macroglossia, nephron–urological malformations, lateralized overgrowth, hyperinsulinism, hypoglycemia, ear anomalies (lobe creases and/or helical pits), nevus flammeus, and organomegaly [9,20].

Nonmalignant renal tract abnormalities occur in 28–61% of BWS patients, and in 87% of the cases are asymptomatic [21,22]. The prevalence of caliceal diverticula, nephrolithiasis, and hydronephrosis is increased compared to that in the general population, and cortical and medullary cysts occur in about 10% of the patients. A minority of nephron–urological anomalies may be severe and require medical or surgical management, such as severe vesicoureteral reflux that could result in kidney damage and recurrent urinary tract infections [9,22,23,24].

#### 3.2.2. Molecular Pathogenesis

Two gene clusters involved in cell cycle progression and somatic growth control, which are controlled by two separate Imprinting Centers (IC1 and IC2) and located in the chromosome region 11p15.5, are associated with BWS. Different methylation patterns of the maternal and paternal alleles characterize IC1 and IC2 [25]. The pathogenesis of BWS involves numerous molecular processes. Loss of methylation at IC2 (IC2-LoM), which results in reduced expression of the cyclin-dependent kinase inhibitor 1 C (*CDKN1C)* gene, that is normally expressed only from the maternal chromosome, accounts for about 50% of the cases. Gain of methylation at IC1 (IC1-GoM), which causes biallelic expression of the insulin growth factor 2 (*IGF2*) gene and diminished expression of the oncosuppressor H19 gene, accounts for 5 to 10% of BWS cases. Mosaic paternal uniparental disomy (UPD), which accounts for 20% of the cases, is characterized by altered expression of both gene clusters. Five to ten percent of the cases have *CDKN1C* gene loss-of-function mutations. Chromosomal rearrangements (i.e., duplications, translocations, inversions, deletions) involving genes in IC clusters, account for less than 1% of all BWS cases. Furthermore, 15% of the patients having a suggestive phenotype have no identifiable genetic abnormalities [20,25,26]. 

#### 3.2.3. Occurrence of Embryonal Tumors

The probability of developing a tumor is generally between 5 and 10% for patients with BWS, with the highest risk occurring in the first two years of life (Table 1) [9,27,28]. A cancer risk gradient distinguishes the four major molecular groups, with IC1-GoM showing the highest risk, followed by segmental UPD(11)pat, *CDKN1C* gene mutation, and IC2-LoM [27,28].

**Figure 2:**
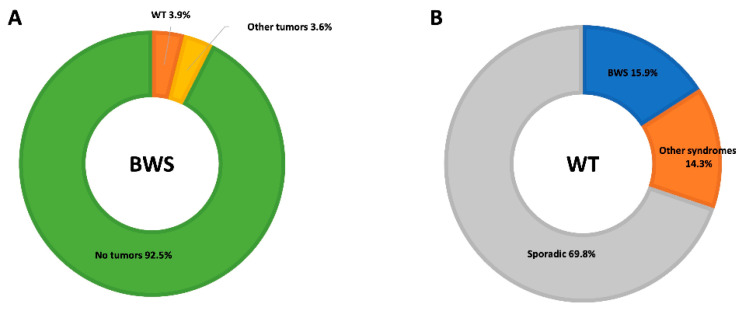
Graphical representation of the frequency of WT in patients with BWS and of BWS in patients with WT. (**A**) Frequency of the occurrence of WT and other tumors in patients with a BWS diagnosis [9]. (**B**) Frequency of BWS and other predisposing syndromes in patients diagnosed with WT [7].

The most common types of embryonal tumors are WT (52% of all tumors), hepatoblastoma (14%), neuroblastoma (10%), rhabdomyosarcoma (5%), and adrenal carcinoma (3%) (Figure 2) [9,27,29]. 

There are also differences in the developed tumor type across molecular subgroups. Specifically, WT are mostly associated with IC1-GoM (95% of the malignancies in this group) and UPD(11)pat. Conversely, patients with *CDKN1C* mutations and IC2-LoM have a low risk of WT (1.4% and 0.2%, respectively) [9,11,27].

### 3.3. Characteristics and Management of WT in BWS Patients

#### 3.3.1. Prevalence

BWS is one of the most common syndromes associated with WT (Figure 2). Nevertheless, the prevalence of BWS in WT patients is underestimated, and WT may be the presenting symptom of an otherwise misdiagnosed syndrome [30].

In a recent study, Hol et al. showed a significant prevalence of BWS (16%) in 126 unselected individuals with WT from a national cohort of children with cancer subjected to a thorough genetic diagnostic analysis between 2015 and 2020 [7]. This is an unexpectedly high frequency if compared with only that of 1–8% in earlier reports [5,6,16,31,32]. The prospective Children’s Oncology Group (COG) study AREN0534 enrolled 34 patients with bilaterally predisposed unilateral WT, and BWS was the most frequent syndrome (26%) diagnosed in these patients [33]. 

#### 3.3.2. Gender and Age at Diagnosis

Several studies have shown that female BWS patients develop WT more frequently than male BWS patients [32,34]. This finding is consistent with the fact that WT is one of the few childhood cancers that affects girls more frequently than boys [35].

In general, patients with WT-associated syndromes tend to be considerably younger at the time of WT onset than those with sporadic WT. This suggests that an early age at diagnosis should raise awareness of an underlying WT-predisposing syndrome [31,32]. However, BWS patients show a higher age at WT diagnosis compared to those with other predisposing conditions, even though it is still slightly lower than that of sporadic WT patients [30,31,32,36].

Welter et al. reported a significantly lower age at diagnosis for patients with *WT1*-mediated syndromes (WAGR, 21 months, DDS, 16 months) compared to sporadic WT patients (39 months) and an only slightly lower age at WT diagnosis in BWS patients (30 months) [32]. Similarly, in a cohort of 12 patients diagnosed with BWS after presenting with a WT, MacFarland et al. reported a median age at WT diagnosis lower than that of children with sporadic WT (26 months and 42 months, respectively) but slightly higher than that of patients with other genetic predisposition conditions [18,30].

Specifically, Porteus et al. reported a younger age at WT diagnosis in BWS patients with bilateral disease if compared to those with unilateral disease [31].

Furthermore, the abdominal ultrasound (US) screening program’s observance may certainly have an impact on the age at which WT individuals are diagnosed. McNeil et al. reported a median age of 16 months at WT diagnosis in a cohort of regularly US-screened BWS patients [36]. 

#### 3.3.3. Staging

In most studies, the tumor stage distribution indicated that WT associated with BWS were more likely to present at an early stage than sporadic WT [9,31,37]. Interestingly, Porteus et al. reported differences in the clinical characteristics of BWS patients treated with two subsequent protocols (NWT3 and NWT4), with a tendency towards smaller tumors in BWS patients only [31]. This trend might be probably explained by an intensification of US screening in children with BWS. Furthermore, Welter et al. reported a significant lower volume calculated from imaging studies at diagnosis in a large cohort of 197 patients with WT-associated syndromes including BWS patients [32]. 

A high incidence of bilateral WT or bilateral disease (WT with NRs or nephroblastomatosis visible on imaging in the contralateral kidney) was reported in syndromic patients if compared to sporadic WT patients (21.2% vs. 7.4%) [32,38,39]. Bilaterality was especially frequent in patients with BWS (31.3% of WT cases), WAGR (30%), and DDS (29.2%) [32]. Consequently, patients with unilateral disease and BWS should always be regarded as predisposed for bilaterality, and in case of bilateral disease, BWS needs to be suspected, if it has not been diagnosed yet [31].

In general, metastatic tumors are significantly less frequent in patients with BWS as compared to non-syndromic WT patients. This may be attributed to an early diagnosis of WT and highlights the value of screening programs even more [31,40].

#### 3.3.4. Histology

Most patients with BWS have intermediate-risk WT according to the SIOP classification after neoadjuvant chemotherapy. A trend towards a higher incidence of blastemal type WT was reported in BWS patients and could be possibly due to the frequently *IGF2*-driven biology [32]. The association with focal and diffuse anaplasia, as reported by Green et al., was not confirmed in further studies [31,41].

A high percentage of WT cases in BWS patients are associated with NRs/nephroblastomatosis [9,31,32,42,43]. NRs are foci of embryonic renal cells that abnormally persist beyond 36 weeks of gestation and are regarded as precursors of WT. NRs are histologically and anatomically classified as either perilobar NRs, if confined to the periphery of the renal lobe, or intralobar NRs, if found anywhere within the renal lobe. The presence of multiple or diffusely distributed NRs is termed nephroblastomatosis [19,44,45,46].

Overall, 30–40% of patients with WT have NRs, whereas this percentage is markedly higher in patients with WT associated with BWS (up to 70–80%) [43,47].

Additionally, BWS-associated WTs tend to harbor perilobar NRs in the adjacent kidney tissue rather than intralobar NRs that are more frequently associated with *WT1*-related WT. These perilobar NRs may even encompass the entire renal cortex in extreme cases [29,43,44,47].

#### 3.3.5. Radiological Diagnosis

The first imaging technique used when a WT is suspected is abdominal US [48]. The two primary cross-sectional modalities are magnetic resonance imaging (MRI) and computed tomography (CT) abdominal scans [49]. MRI is currently recommended as the primary imaging method for evaluating renal tumors by the Renal Tumor Committee of the COG and the International Society of Paediatric Oncology Renal Tumors Study Group (SIOP-RTSG) [49].

MRI is recommended for a better assessment of potential NRs and their distinction from malignant tumors. In BWS patients who frequently exhibit NRs/nephroblastomatosis, differentiating these lesions from WT at diagnosis and during follow-up is crucial [49]. In this context, the most useful MRI sequences are Diffusion-Weighted Imaging (DWI) and contrast-enhanced T1-weighted imaging. Indeed, NRs are best visualized on T1-weighted imaging, with a hypo-intense appearance and a homogeneous signal after contrast [50,51,52,53].

On the contrary, CT is not a primary indication due to its main drawbacks: a low specificity for distinguishing NRs from WT and radiation exposure, which must be avoided especially in syndromic patients [54]. 

#### 3.3.6. Role of Biopsy

Most malignant childhood tumors are primarily biopsied to be histologically characterized and further managed with the appropriate treatment. The significantly higher prevalence of WT among childhood kidney tumors renders it unnecessary to perform a biopsy in most cases [55,56]. 

According to the SIOP-RTSG guidelines, patients older than 6 months and with a WT-typical radiological presentation should receive a preoperative chemotherapy without histological confirmation [55,57]. The approach of preoperative chemotherapy aims to reduce the risk of tumor rupture during surgery and allow for the stratification of postoperative chemotherapy intensity and duration based on the response to the preoperative treatment [58]. 

Based on literature evidence, the SIOP-RTSG recommends that a diagnostic biopsy should be conducted when either the clinical or the radiologic presentation is considered atypical for WT and therefore does not allow initiating a “presumptive” neoadjuvant chemotherapy [50,59,60,61]. A most challenging situation occurs with children older than 10 years, for whom the relative likelihood of a non-WT diagnosis is increased [55,59,62].

The strategy recommended by the COG also does not advocate an initial biopsy, since a primary nephrectomy is indicated, when feasible, followed by adjuvant therapy based on stage and molecular and histological risk factors. 

In BWS patients, WT usually occurs in younger children. The challenge, in this case, is not the differentiation from other non-WTs but rather the possibility of distinguishing small tumors from NRs. It is important to highlight that there are no consistent histological, immunohistochemical, or molecular characteristics that can differentiate hyperplastic NRs from a WT by pathologic examination. Consequently, the analysis of tissues collected through biopsy is discouraged as a standard diagnostic method. As the radiological MRI pattern is usually very suggestive, presumptive chemotherapy can be started with relative confidence based on imaging patterns [63]. 

#### 3.3.7. Treatment Recommendations for Children with WT and BWS

##### Neoadjuvant and Adjuvant Chemotherapy

Neoadjuvant chemotherapy for patients with bilateral disease or for those with unilateral WT but bilaterally predisposed disorders such as BWS is the approach recommended by both the SIOP and the COG groups [33,45,58,64]. One major goal of preoperative chemotherapy, even in the presence of a monolateral tumor, is to obtain tumor shrinkage to maximize nephron-sparing surgery (NSS) opportunities. The possibility of a contralateral metachronous tumor must be considered, and avoiding radical nephrectomy must be prioritized (once that the surgical oncological outcome is guaranteed). To this aim, an approach using carboplatin–etoposide in patients with an unsatisfactory response after a more conventional two-drug chemotherapy is under evaluation [58]. 

Several studies reported an excellent WT response to preoperative chemotherapy in BWS patients [1,32,33,58,64]. In the perspective COG AREN0534 study, the initial induction therapy included vincristine and dactinomycin if no biopsy was performed and imaging revealed local disease only. Vincristine, dactinomycin, and doxorubicin were administered in case of metastatic disease and if no biopsy was performed. More intense regimens were used in case of anaplastic histology at biopsy [33]. 

Welter et al. documented that BWS patients showed a significant volume reduction of 86.9% after neoadjuvant chemotherapy. In contrast, in other patients with WT-predisposing syndromes included in the same cohort such as DDS, no real change of tumor volume under preoperative chemotherapy was observed. This finding can be explained by the frequent stromal histology seen in DDS patients, which is the primary reason for the failure of response to preoperative chemotherapy [32]. 

Adjuvant postoperative treatment guidelines based on histological types and local stage follow the same principle as for WT tumors in patients without predisposing syndromes. At the end of treatment in case of nephroblastomatosis in one or both sides, a maintenance regimen including vincristine and dactinomycin could be indicated to prevent or reduce the incidence of a metachronous WT [58,65]. 

##### Nephron-Sparing Surgery

In unilateral sporadic WT arising in children without known cancer predispositions, radical nephrectomy represents the gold standard, whereas NSS (strongly recommended for bilateral disease) is performed only in selected cases, better if following shared guidelines. We know that significant expertise in NSS is crucial to maximize the benefits and to lessen the related risks [66]. 

The reasons to consider NSS in BWS patients also with unilateral WT are several. BWS patients show a significant increased risk of developing a bilateral or metachronous WT, which is up to 8.6% [39]. Children with perilobar rests, the most common type of NRs occurring in children with BWS, have approximately a six-fold risk of metachronous WT when compared with children without NRs. This is particularly true for children younger than 12 months [39]. Another reason for considerate NSS in BWS patients is that more than 25% of BWS patients have non-malignant renal anomalies that may potentially lead to later complications and renal function impairment [9,21]. We here remind that BWS patients usually have tumors with considerably smaller volume than children with sporadic WT as a result of US screening [37], and smaller tumors are amenable to NSS assuming that they do not involve hilar or vascular structures.

The benefits of NSS have been demonstrated in several cohorts of syndromic patients including BWS patients with unilateral WT [33,34,36,64,67,68,69]. 

Interestingly, a retrospective US screening in a small cohort of eight patients with BWS who underwent radical nephrectomy showed that all children would have been candidates for NSS considering that the tumor was confined, with at least two-thirds of the kidney free of tumor, and no involvement of hilar or vascular structures was documented [36]. In a retrospective French cohort of 34 patients including 24 cases with BWS-associated unilateral WT treated according to the SIOP protocols, NSS after preoperative chemotherapy was performed in 9 patients, while 25 patients underwent radical nephrectomy. Particularly, 89% (8/9) of the patients who underwent NSS were BWS patients. No recurrence was noticed among the patients who received NSS [34]. Furthermore, Romao et al. reported excellent outcomes in a retrospective review of a cohort of eight patients with WT-predisposing syndromes including BWS treated for unilateral WT. NSS was performed in six patients, and no recurrences were documented [69].

Additionally, Ehrlich et al. reported the results of a prospective COG AREN0534 study including 34 patients (9 BWS patients) with multicentric or bilaterally predisposed unilateral WT treated with a standardized approach of preoperative chemotherapy to facilitate NSS in lieu of radical nephrectomy. Overall, pre-nephrectomy chemotherapy allowed NSS in 65% of the patients. Almost all (89%; 8/9) BWS patients underwent preservation of the renal parenchyma. The four-year event-free survival (EFS) and overall survival (OS) rates were 94% and 100%, respectively. There were only two events, one of which was a local relapse in a child with BWS that occurred 18 months after the end of treatment [33,64] (Table 2).

#### 3.3.8. Outcome

Vaughan et al. indicated that BWS confers a favorable prognosis after reporting a 100% EFS in 10 patients with WT and BWS [70]. Differently, more recent studies reported similar EFS and OS rates for WT-with-BWS and sporadic WT patients [18,31,34]. 

Welter et al. reported a significant worse EFS and increased incidence of relapse in BWS patients compared to patients with other cancer-predisposing syndromes, without significant differences in OS. Specifically, 5 out of 22 (23%) BWS patients with unilateral WT relapsed, of whom three showed metachronous disease (3, 4.5, and 6 years after the initial diagnosis). Of the other two relapsed patients, one patient developed a local relapse in the same kidney, and the other developed lung metastasis without a local or metachronous tumor, and both were alive at the time of that study report. Furthermore, EFS tends to be worse for all patients with concomitant nephroblastomatosis, particularly if they had developed WT [32].

#### 3.3.9. Screening/Surveillance

The benefit of routine screening for WT in the BWS population has largely been supported [9,11,27,28,29,37,40,71]. The purposes of the early detection of WT by surveillance are likely to enable early NSS, allow a less-intensive (that is, less toxic) chemotherapy, and avoid radiotherapy [31,33,34,72,73]. Currently, a renal US examination is the preferred modality to screen for WT. The doubling time of WT cells has been estimated to be 11–13 days, and US examination is recommended every 3–4 months [36,37,74]. Patients should be screened until 7–8 years of age, as the majority of WT in BWS patients will have developed by this time [9,31]. The International BWS Consensus Group agreed that tumor surveillance should be targeted to those patients with molecular subgroups of BWS that are at the highest risk (IC1-GoM and segmental UPD(11)pat). Children with BWS associated with IC2-LoM should not be offered routine US examination. Other BWS molecular subgroups and patients with classical BWS and no detectable molecular anomalies should be offered abdominal US screening every 3 months until the age of 7 years [9]. Conversely, the American Association for Cancer Research (AACR) Children Cancer Predisposition Workshop adopted a 1% risk threshold for surveillance and therefore recommended abdominal US for all cases of BWS [29].

#### 3.3.10. Long-Term Renal Complications

Renal impairment is significantly more likely to occur in patients with syndromic WT. The whole renal tissue in individuals with WT-predisposing disorders has molecular abnormalities that raise the risk for the onset of bilateral WT, requiring the surgical removal of a sizeable amount of renal functional parenchyma.

Specifically, BWS does not appear to have an intrinsic propensity to predispose for renal impairment, even if non-malignant renal abnormalities that may cause chronic kidney disease due to recurrent infection or severe reflux and medullary sponge kidney have been described [75,76]. Moreover, nephrotoxic therapies administered as subsequent surgical treatments, chemo-, or radiotherapy may accelerate the occurrence of renal impairment. 

Thus, long-term renal function monitoring is strongly recommended in BWS patients after treatment of WT.

## 4. Conclusions

Overall, the literature data suggest that children with BWS and WT exhibit lower-stage and less metastatic tumors, supporting the benefit of surveillance for WTs in these individuals.

Importantly, WT may be the presenting symptom of BWS characterized by only subtle overgrowth features or silent phenotypes. The awareness of red flags for BWS, such as bilateral or multicentric unilateral disease, and/or the presence of NRs, and/or some mild overgrowth features should raise the suspicion of BWS, and the patient should quickly be referred for genetic testing.

The use of preoperative chemotherapy is always strongly recommended to maximize the possibility of NSS, even in the presence of unilateral tumors. NSS is particularly preferred for patients with BWS, given their significant chance of having both kidneys involved by WT and the fact that around 20% of them develop bilateral disease at some time in the course of the disease (synchronous bilateral disease at diagnosis or metachronous recurrence after initial presentation with unilateral disease). For this purpose, affected patients need to be referred to experienced surgeons and to high-volume surgical centers for NSS.

The early identification of BWS when WT has already occurred, especially if unilateral, is urgent in a temporal sequence of actions, because it can lead to immediate changes in first-line cancer therapy with the goal of expanding indications for renal preservation surgery.

## Figures and Tables

**Table 1:** BWS molecular defect categories and related risk of developing embryonal tumors.

	Overall	IC1-GoM	UPD	*CDKN1C* Mutation	**IC2-LoM**
Malignant tumors	5–10%	28%	16%	6.9%	2.6%
Wilms tumor	3.5%	24%	7.9%	1.4%	0.2%
Hepatoblastoma	1.7%	-	3.5%	-	0.7%
Neuroblastoma	0.7%	0.7%	1.4%	4.2%	0.5%
Rhabdomyosarcoma	0.5%	-	0.3%	-	0.5%
Adrenal carcinoma	0.4%	-	1.1%	-	-

Adapted from [9,11,27,28].

**Table 2:** Selected studies focusing on Nephron-Sparing Surgery in BWS patients with bilaterally predisposed unilateral WT.

	Romao et al., 2012 [69]	Scalabre et al., 2016 [34]	Ehrlich et al., 2020 [33]
Type of study	Retrospective	Retrospective	Prospective
Cohort	8 pts (3 BWS)	34 pts (24 BWS)	34 pts (9 BWS)
Gender (female/male)	-	22/12	21/13
Median age at diagnosis (months)	15	24	33
US surveillance	75%	94%	38%
Preoperative CT	62,5%	97%	100%
Histology	-	28 IR, 5 HR	13 LR, 15 IR, 6 HR
NSS (%)	62,5)	26%; BWS: 33%	62,5%; BWS: 89%
TN (%)	3/8 (37,5%)	74%; BWS 67%	37,5%; BWS: 11%
OS	100% (3 y)	-	100% (4 y)
EFS	100% (3 y)	92.3% (3 y)	94% (4 y)

US: ultrasound, CT: chemotherapy, NSS: nephron-sparing surgery, TN: total nephrectomy, OS: overall survival, EFS: event-free survival.
